# Psychosocial impact of pediatric long COVID: a dyadic analysis of persistent symptoms, sleep, and self-esteem in parents and children

**DOI:** 10.3389/fpsyg.2026.1769305

**Published:** 2026-02-06

**Authors:** Per Ertzgaard, Anneli Wärdig, Karel Duchen, Charlotte Angelhoff

**Affiliations:** 1 Department of Health, Medicine and Caring Sciences, Linköping University, Linköping, Sweden; 2 Clinical Department of Rehabilitation Medicine in Linköping, Region Östergötland, Linköping, Sweden; 3 Crown Princess Victoria Children’s Hospital, Region Östergötland, Linköping, Sweden; 4 Department of Biomedical and Clinical Sciences, Linköping University, Linköping, Sweden; 5 Clinical Department of Allergy Center in Linköping, Region Östergötland, Linköping, Sweden

**Keywords:** chronically ill children, family dynamics, insomnia, long COVID, parental responsibility, sleep quality

## Abstract

**Background:**

Pediatric long COVID can lead to persistent symptoms that affect the child’s daily functioning and may influence family dynamics. Parents of children with chronic conditions may be at risk of experiencing challenges related to their own health, sleep, and self-esteem. Exploring the parent–child dyad may provide a deeper understanding of how long COVID impacts both individuals and their relationship. The aim of this study was to describe health, sleep quality, insomnia symptoms, and self-esteem in parents of children with long COVID, and to see how these factors are affected by the child´s disability exploring child-parent dyads and triads.

**Methods:**

This cross-sectional study, part of the interdisciplinary project POCOKIDS, included 35 parents and 26 children who completed questionnaires on long COVID symptoms, sleep quality, insomnia, and self-esteem.

**Results:**

Parents with persistent symptoms reported poorer sleep, higher insomnia scores, and greater worry about finances, employment, social life, and their child’s education than those without symptoms. Notably, parents without persistent symptoms reported lower self-esteem. Most children reported poor sleep quality, and nearly half met criteria for insomnia symptoms, with girls experiencing more sleep-related difficulties than boys. Children’s self-esteem was less affected than their parents’.

**Discussion:**

The findings reveal a shared psychosocial burden and underscore the need for individualized support addressing both children’s and caregivers’ health and emotional needs.

## 1 Introduction

Long COVID, also known as post-acute COVID-19 syndrome, is a medical condition characterized by persistent long-term symptoms that arise after acute COVID-19 infection. Children with long COVID often suffer from fatigue, breathing problems, headaches, and cognitive issues that impact daily life and social interactions. Many also face psychological challenges like anxiety and mood swings due to prolonged illness (Basaca et al., 2025).

Many pediatric cases remained undiagnosed until a clinical case definition for post COVID-19 condition in children and adolescents was published in February 2023 (World Health Organization, 2023). Even after that, numerous cases remained unreported, with parents frequently citing a lack of awareness of the condition among healthcare providers. They report struggling not only to care for their sick child themselves but also to access healthcare for this previously unknown condition (Angelhoff et al., 2024; Faux-Nightingale et al., 2023; Torres et al., 2024). Children and adolescents with long COVID, along with their parents, have described a “double invisibility,” where their condition is both socially and medically unrecognized, highlighting how the lack of recognition, enduring stereotypes, and poor communication about symptoms can further isolate conditions marked by fatigue and pain (Angelhoff et al., 2024; Lillieberg et al., 2025; Wild et al., 2024). While direct data on the risk of long COVID in parents and children within the same family is scarce, some studies suggest that both groups are affected by the pandemic’s stressors (Thomson et al., 2023). Persistent symptoms in parents themselves, such as fatigue and pain, may further complicate caregiving and family functioning, making it important to consider parental health alongside child outcomes. Health, sleep, and self-esteem are fundamental to parents’ ability to recover and provide care, and these domains are often compromised by chronic illness and prolonged stress.

In order to seek medical care for their own problems, care for their sick child, and manage everyday life, parents ought to prioritize their own health, as high stress levels and worries about the child’s health may result in sleep deprivation, which in turn may impair their ability to provide adequate care (Angelhoff et al., 2018; Meltzer and Moore, 2008). Additionally, parental anxiety and stress have been found to mediate the relationship between child anxiety and sleep problems (Francazio et al., 2015). Smith et al. (2022a,b) describe parents’ sleep when their child is ill as being influenced by multiple interrelated factors, including sleep onset time, sleep disturbances, and sleep duration. These aspects are further affected by the child’s condition, the parents’ sleeping environment, coping strategies, available support resources, and the parents’ overall health. Together, these factors interact and influence parents’ biopsychosocial well-being.

No previous studies are found specifically focused on self-esteem in parents of sick children. It is though suggested that caring for a child with a chronic illness can profoundly impact parents’ self-esteem. The emotional strain, feelings of helplessness, and constant worry experienced by caregivers of children with various disorders, diminish their sense of accomplishment and control (Unvanli et al., 2023; Wang and Anderson, 2018). This phenomenon is likely to apply to parents of children with long COVID, where uncertainty, lack of medical recognition, and fluctuating symptoms may exacerbate emotional and psychological burdens, ultimately impacting their self-esteem and overall well-being. Furthermore, the inability to fully meet their child’s needs or to prevent the child’s suffering can evoke feelings of guilt and inadequacy, which are frequently associated with lower self-esteem (Budiarto and Helmi, 2021).

Haddad et al. (2022) report that prolonged symptoms tend to cluster within families, suggesting that family-level interventions for long COVID could be beneficial. Pediatric long COVID places significant strain on parents, yet there is a notable lack of research on how this condition affects their health, sleep, and self-esteem. Given that these domains are essential for coping and caregiving, and may be influenced not only by the child’s illness but also by persistent symptoms in parents themselves, they need to be studied further. Addressing these gaps is crucial to improve the well-being of both parents and children.

The aim of this study was to describe health, sleep quality, insomnia symptoms, and self-esteem in parents of children with long COVID, and to see how these factors are affected by the child´s disability exploring child-parent dyads and triads.

## 2 Method

### 2.1 Design

This study employed quantitative cross-sectional design. The study was integrated into the larger interdisciplinary research project “the POCOKIDS study” (POstCOvid in KIDS study), which aims to explore the biological, physiological, psychological, and social impacts of long COVID on children and their families in Sweden (Angelhoff et al., 2024; Lillieberg et al., 2025).

### 2.2 Participants and procedure

Children aged 6 to 18 years, referred to the Pediatric Rehabilitation Clinic in Region Östergötland and the Pediatric and Adolescent Medical Outpatient Clinics in Region Jönköping County, showing clinical symptoms consistent with long COVID (such as muscular and cognitive fatigue, palpitations, and recurrent headaches) at least 8 weeks after the onset of a COVID-19 infection, along with their parents, were included in the study. Exclusion criteria were families who did not speak the Swedish language and parents who did not have legal custody of the child.

The child was called for a general pediatric examination of their symptoms at one of the clinics mentioned above following the referral. A pediatrician assessed whether the child met the criteria for inclusion in the POCOKIDS study and provided written information about the study. A research nurse then contacted the family, gave oral information about the study, and inquired about their willingness to participate. If the family agreed, both the child and parent were scheduled for an inclusion visit, where additional information was provided, and consent forms were signed.

During one of the child’s scheduled study visits, the child and the accompanying parents were given a personal code and a link to the questionnaire, which was distributed via the Webropol survey tool. They had the option to complete the questionnaire either during the visit or at home. If one parent was not present during the visit, the attending parent was provided with a link to allow the non-accompanying parent to participate in the survey. Data collection spanned from October 2022 to December 2023. As the study used consecutive sampling of eligible participants during the study period, no formal sample size calculation was performed.

### 2.3 Measures

#### 2.3.1 Study-specific questionnaire/persisting symptoms after COVID-19

The parents completed a study-specific questionnaire, which included demographic data such as age, gender, employment status, marital status etc., as well as questions regarding their own COVID-19 infection. The parents were also asked about their own persistent symptoms after COVID-19, based on data from the Linköping COVID-19 Study (Divanoglou et al., 2021). The options included: no lingering symptoms, shortness of breath, extreme fatigue (physical and mental), fever, altered sense of smell or taste, headache, elevated resting heart rate or palpitations, cognitive impairment (e.g., memory and concentration difficulties), gastrointestinal issues, muscle weakness, neurological symptoms (e.g., numbness), mental health issues (e.g., depression, anxiety), pain (e.g., chest pain, muscle or joint pain), and sleep disturbances. Moreover, the questionnaire included a question about their perceived general health on a scale from 1 (very poor) to 5 (very good) and questions about concerns related to their child’s illness.

#### 2.3.2 Pittsburgh sleep quality index

To assess sleep quality in both parents and children, Pittsburgh Sleep Quality Index (PSQI) was used. The PSQI consists of 19 self-rated items that generate seven component scores: subjective sleep quality, sleep latency, sleep duration, habitual sleep efficiency, sleep disturbances, use of sleeping medication, and daytime dysfunction. Each component is scored on a scale from 0 to 3, with higher scores indicating poorer sleep. The seven component scores are summed to yield a global score ranging from 0 to 21. The cut-off for poor sleep quality is 5 or higher (Buysse et al., 1989). The PSQI is widely used in sleep research and has been validated in both adult and adolescent populations (Buysse et al., 1989; de la Vega et al., 2015; Raniti et al., 2018), as well as in studies on COVID-19 (Souza et al., 2021).

#### 2.3.3 The insomnia severity index

The Insomnia Severity Index (ISI) was used to assess insomnia symptoms. The ISI is a validated tool for assessing the severity of insomnia. It consists of seven items, each rated on a scale from 0 to 4, yielding a total score ranging from 0 to 28. Scores between 8 and 14 indicate mild insomnia, 15 to 21 indicate moderate insomnia, and 22 to 28 indicate severe insomnia. A cut-off score of ≥10 is used to identify clinically significant insomnia symptoms (Bastien et al., 2001). The ISI has previously been used in studies involving both adults and adolescents (Cerri et al., 2023), and in studies on COVID-19 (AlRasheed et al., 2022).

#### 2.3.4 Rosenberg self-esteem scale

Self-esteem was measured using the Rosenberg Self-Esteem Scale (RSE), which consists of 10 statements answered on a 4-point Likert scale (1 = Strongly disagree, 4 = Strongly agree). The total score ranges from 10 to 40 (Rosenberg, 1989). Based on previous studies (Angelhoff et al., 2021; Isomaa et al., 2013; Lundberg et al., 2018, 2019), the following cut-off scores for self-esteem were used: 10–24 indicates low self-esteem, 25–35 represents normal self-esteem, and 36–40 indicates high self-esteem. The Swedish version of the RSE has been evaluated for psychometric properties in Sweden and demonstrated good internal consistency, criterion validity, convergent and discriminant validity, and sensitivity to change (Eklund et al., 2018).

### 2.4 Statistical analysis

Descriptive statistics, e.g., means, standard deviations (sd), medians (md), quartiles (q1–q3), frequencies (n) and percentages (%) were used to describe participants’ demographic characteristics, parent’s symptoms, and parent and child ratings of sleep quality (PSQI), insomnia symptoms (ISI) and self-esteem (RSE). Normal distribution was assessed using the Kolmogorov–Smirnov test. Since the data were not normally distributed, non-parametric tests were employed. A *p*-value of <0.05 was considered statistically significant.

The PSQI scores were analyzed by summing the scores from the seven components to obtain a total score representing sleep quality (Buysse et al., 1989). The ISI scores were summed up to provide a total insomnia severity score. In the RSE, negatively worded items (Items 3, 5, 8, 9, and 10) were reverse-coded (e.g., 4 → 1, 3 → 2, etc.) prior to computing the total self-esteem score. Missing values were replaced with the mean value for the entire population to avoid excluding any data points (Field, 2024).

The Mann–Whitney U test was used to compare PSQI, ISI and RSE scores between mothers and fathers and between boys and girls. Wilcoxon’s signed rank test was used to compare parent’s reported scores on PSQI, ISI and RSE with their child’s scores on the same instruments, i.e., paired child–parent dyads.

To examine the associations between self-reported symptoms and the total scores on the PSQI, ISI, and RSE, point-biserial correlations were conducted. As the symptom variables were dichotomous and the scale scores were treated as continuous, Pearson’s r_pb_ was used.

## 3 Results

### 3.1 Participant characteristics

The study included 35 parents, of whom 13 (37%) were men, and 26 children, of whom nine (35%) were boys. In nine families, both parents participated. One child withdrew from the study, but the mother from that family chose to continue her participation. The parents’ mean age was 47.4 years (SD 5.6), and the children’s mean age was 14.0 years (SD 2.8). Demographic data and questionnaire results by parent and child gender are shown in Table 1.

**Table 1:** Participant demographics and mean scores on PSQI, ISI, and RSE by parent and child gender.

	Mothers (*n* = 22)	Fathers (*n* = 13)	Girls (*n* = 18)	Boys (*n* = 8)
Mean age (SD)	47.0 (5.1)	59.6 (5.8)	14.8 (3.1)	12.9 (1.9)
Single parent, *n* (%)	5 (22.7)	–	–	–
On sick leave, *n* (%)	6 (27.3)	0 (0)	–	–
Perceived general health, *n* (%)	2.7 (1.3)	1.8 (0.99)	–	–
PSQI, mean (SD)	9.0 (4.6)	6.2 (4.4)	9.8 (4.5)	6.1 (1.6)
ISI, mean (SD)	11.2 (6.4)	8.6 (7.9)	10.9 (6.7)	6.0 (3.9)
RSE, mean (SD)	18.6 (8.7)	15.5 (5.6)	23.9 (6.3)	22.1 (5.5)

Perceived general health (only parents): 1–5 (1 = very poor, 5 = very good). PSQ, Pittsburgh Sleep Quality Index (0–21), cut-off ≥5 indicates poor sleep quality. ISI, Insomnia Severity Index (0–28), cut-off >10 indicates clinically significant insomnia (8–14 mild, 15–21 moderate, 22–28 severe). RSE, Rosenberg Self-Esteem Scale (10–40), 10–24 low, 25–35 normal, 36–40 high self-esteem. PSQI and ISI were completed by 16 girls and 7 boys.

### 3.2 Parents’ health

**Figure 1:**
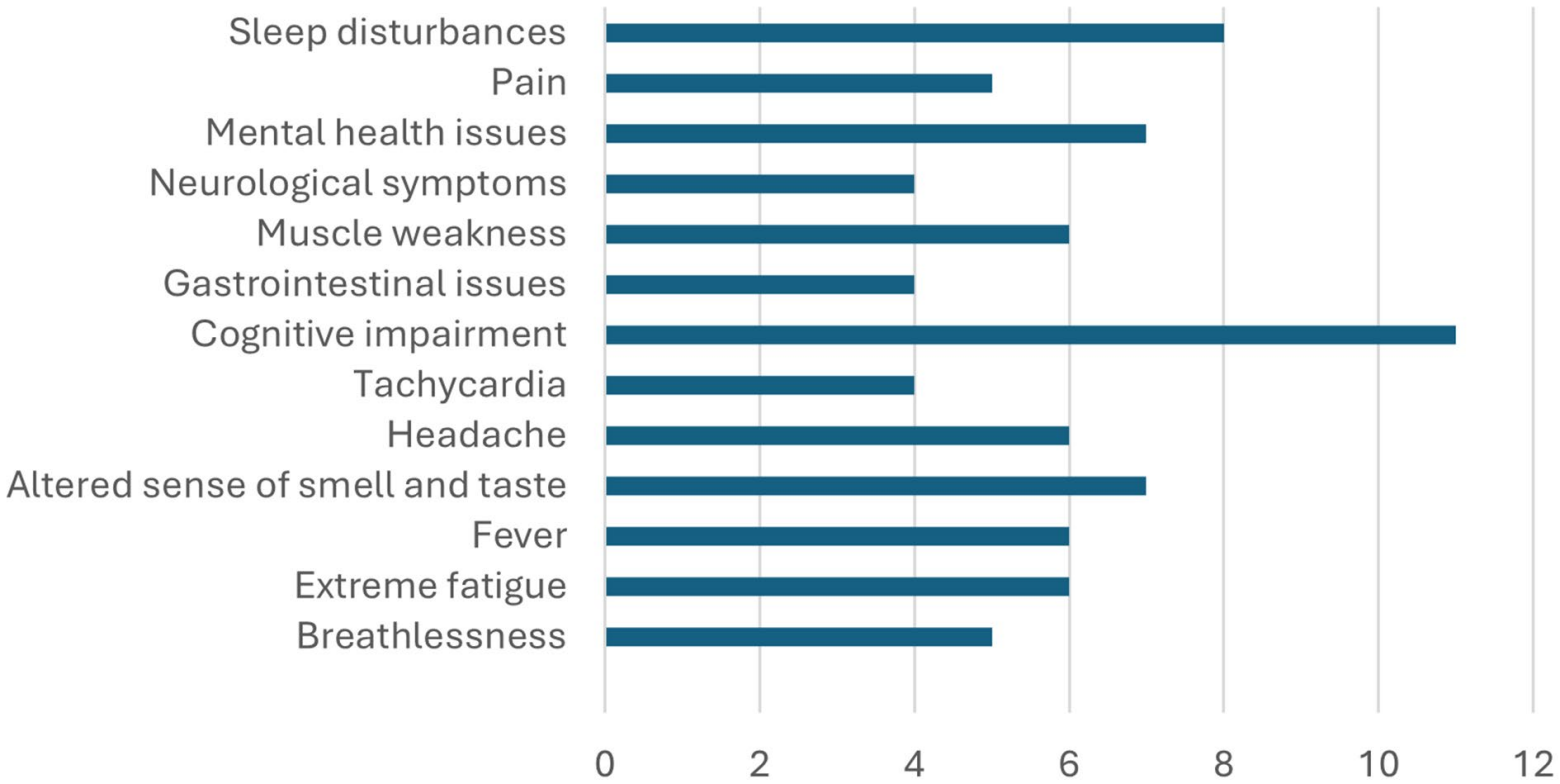
Number of parents reporting persistent symptoms after COVID-19 infection (*n* = 31).

Of the 35 parents, seven parents (20%) rated their general health status as poor or very poor. The mean self-reported health rating was 2.4 (SD = 1.2). Twenty-five parents (71%) reported having tested positive for COVID-19. Six parents (17%) believed they had contracted COVID-19 but were not tested, while four parents (11%) stated that they had neither been tested nor infected. Thirty-one parents (89%) responded to the questionnaire about their own symptoms. Of these, 14 parents (40%), whereof 12 mothers and two fathers, reported persistent symptoms following their own COVID-19 infection, where cognitive impairment and sleep disturbances were the most common symptoms (see Figure 1). Eight mothers and one father reported that they had sought medical care for their persistent symptoms.

### 3.3 Parental concerns related to their child’s illness

All of the parents reported one ore more concerns related to their child’s illness, where “worry about child’s education” and “frequent or constant worry about the future” was the most frequent (Table 2). Significant correlations were found between persistent symptoms and concern about financial situation (Pearson *r*_pb_ 0.460, *p* = 0.005), employment-related anxiety (Pearson *r*_pb_ 0.432, *p* = 0.01), concerns related to social life (Pearson *r*_pb_ 0.477, *p* = 0.004), and worry about child’s education (Pearson *r*_pb_ 0.421, *p* = 0.012). No significant correlation was found between persistent symptoms and worry about the future.

**Table 2:** Parents’ concerns related to the child’s illness (*n* = 35).

Parental concerns	*n* (%)
Concerns about the consequences of their child’s COVID-19 infection	34 (97%)
Worry about child’s education	24 (69%)
Frequent or constant worry about the future	16 (46%)
Concern about financial situation	10 (29%)
Employment-related anxiety	9 (26%)
Concerns related to social life	6 (17%)

### 3.4 Sleep quality and insomnia symptoms

Among parents (*n* = 35), the median PSQI score was 7.0 (IQR = 7, mean = 7.9, SD = 4.7). A total of 26 parents (74%) scored above the cut-off for poor sleep quality (≥5), including 13 parents reporting no persistent symptoms. However, parents reporting persistent symptoms had poorer sleep quality than those without (*p* = 0.01). The median ISI score was 9.0 (IQR = 10, mean = 10.3, SD = 7.0). Seventeen parents (49%) scored above the cut-off for clinically significant insomnia symptoms (≥10), including eight parents reporting no persistent symptoms. Parents reporting persistent symptoms scored higher for insomnia (*p* = 0.03). No significant difference was found in PSQI or ISI between mothers and fathers.

Among children (*n* = 22), the median PSQI score was 7.5 (IQR = 6, mean = 8.8, SD = 4.6). All responding children except for one 15-year-old boy, scored above the cut-off indicating poor sleep quality (*n* = 21, 95%). The median ISI score among children was 8.0 (IQR = 9.3, mean = 9.5, SD = 6.4). Ten children (45%) scored above the cut-off for clinically significant insomnia symptoms. There was a significant difference between girls and boys in both PSQI (*p* = 0.01) and ISI (*p* = 0.03), indicating that girls have more sleep related symptoms.

No statistically significant differences were found between parents’ and their children’s scores on PSQI (*p* = 0.70) or ISI (*p* = 0.45) when comparing paired child–parent dyads.

### 3.5 Self-esteem

Among parents (*n* = 35), 30 reported low self-esteem (≤ 24). Ratings for the total RSE mean score showed a median of 14.6 (IQR = 9.6, mean = 17.4, SD = 7.8). There was a difference (*p* = 0.04) between parents reporting persistent symptoms (median 19.5) and those who reported no persistent symptoms (median 13.6), indicating higher self-esteem in parents with persistent symptoms.

Children’s ratings (*n* = 25) for the total RSE mean score showed a median of 23.3 (IQR = 10.3, mean = 23.2, SD = 5.9), which falls just below the cut-off for low self-esteem, whereof eight children scored normal to high self-esteem. No statistically significant difference was found between girls and boys (*p* = 0.44).

A statistically significant difference (*p* = 0.004) was found in RSE scores between parents and their children, with children reporting higher self-esteem.

### 3.6 Correlations between persistent symptoms and sleep, insomnia, and self-esteem in parents

Significant correlations were observed between parents reporting persistent symptoms and higher scores on PSQI (Pearson *r*_pb_ 0.410, *p* = 0.015), ISI (Pearson *r*_pb_ 0.427, *p* = 0.011), and RSE (Pearson *r*_pb_ 0.446, *p* = 0.007), see Table 3.

**Table 3:** Point-biserial correlations (Pearson *r_pb_*) between subjective parental persistent symptoms versus Pittsburgh Sleep Quality Index (PSQI), the Insomnia Severity Index (ISI) and the Rosenberg Self-Esteem Scale (RSE).

Symptoms	Statistics	PSQI	ISI	RSE
Persistent symptoms	Pearson *r*_pb_	0.410^*^	0.427^*^	0.446^**^
	*p*-value	0.015	0.011	0.007
Breathlessness or difficulty breathing (dyspnea)	Pearson *r*_pb_	0.257	0.434^*^	0.596^**^
	*p*-value	0.163	0.015	<0.001
Extreme fatigue (physical and mental)	Pearson *r*_pb_	0.310	0.370^*^	0.496^**^
	*p*-value	0.090	0.041	0.005
Fever or feeling feverish	Pearson *r*_pb_	0.196	0.310	0.469^**^
	*p*-value	0.292	0.089	0.008
Altered sense of smell and taste	Pearson *r*_pb_	0.234	0.318	0.393^*^
	*p*-value	0.206	0.082	0.029
Headache	Pearson *r*_pb_	0.405^*^	0.346	0.616^**^
	*p*-value	0.024	0.057	<0.001
High resting heart rate or palpitations (tachycardia)	Pearson *r*_pb_	0.266	0.516^**^	0.601^**^
	*p*-value	0.148	0.003	<0.001
Cognitive impairment, e.g., memory and concentration difficulties	Pearson *r*_pb_	0.343	0.323	0.501^**^
	*p*-value	0.059	0.076	0.004
Gastrointestinal issues	Pearson *r*_pb_	0.199	0.150	0.509^**^
	*p*-value	0.284	0.421	0.003
Muscle weakness.	Pearson *r*_pb_	0.291	0.442^*^	0.611^**^
	*p*-value	0.112	0.013	<0.001
Neurological symptoms, e.g., numbness.	Pearson *r*_pb_	0.333	0.516^**^	−0.028
	*p*-value	0.067	0.003	0.883
Mental health issues, e.g., depression, Anxiety, or low mood.	Pearson *r*_pb_	0.396^*^	0.588^**^	0.571^**^
	*p*-value	0.028	<0.001	<0.001
Pain, e.g., chest pain or muscle and joint pain.	Pearson *r*_pb_	0.257	0.434^*^	0.596^**^
	*p*-value	0.163	0.015	<0.001
Sleep disturbances.	Pearson *r*_pb_	0.580^**^	0.521^**^	0.591^**^
	*p*-value	<0.001	0.003	<0.001

*Correlation is significant at the 0.05 level (2-tailed). **Correlation is significant at the 0.01 level (2-tailed).

## 4 Discussion

This study provides novel insights into the health, sleep quality, insomnia symptoms, and self-esteem of parents of children with long COVID, framed within the parent–child dyad. The findings reveal persistent symptoms, significant sleep disturbances, moderate levels of insomnia, and notably low self-esteem among parents, underscoring the psychosocial toll of caregiving in the context of pediatric long COVID. This discussion highlights three main findings: a substantial proportion of parents report persistent symptoms; parents of children with long COVID experience pronounced sleep problems and low self-esteem; and parents with persistent symptoms are associated with worse sleep but higher self-esteem.

Although both children and parents reported similarly poor sleep quality and insomnia symptoms at the group level, these sleep outcomes did not correlate within dyads, indicating that sleep difficulties were influenced by individual rather than shared family factors. In contrast, self-esteem was significantly less affected in children compared to their parents.

First, an important and under-discussed issue in literature is the co-occurrence of long COVID within families. In this study, 40% of parents reported persistent symptoms, including fatigue, cognitive impairment, tachycardia, and pain—conditions that significantly correlated with sleep disturbances and insomnia. This adds a vital layer of complexity: not only are these parents managing their child’s condition, but many are doing so while symptomatic themselves. These findings align with Haddad et al. (2022), who showed clustering of persistent symptoms within families, and suggest a bidirectional health burden within the household. The burden of unresolved physical symptoms, especially when unrecognized or inadequately treated, may amplify feelings of helplessness, guilt, and fatigue in parents (Khoreva, 2022; Zheng et al., 2023).

Second, parents of children with long COVID reported substantial sleep disturbance and psychological burden. Our finding that 74% of parents had poor sleep quality and 49% scored above the threshold for clinically significant insomnia symptoms aligns with emerging data showing widespread sleep disturbances during and following the COVID-19 pandemic. Moreover, more than half of the mothers in our sample reported persistent symptoms, which may have further compromised their sleep and contributed to increased caregiving strain. AlRasheed et al. (2022) reported high global rates of insomnia during the pandemic, with caregivers of chronically ill individuals being particularly vulnerable. Similarly, studies involving parents of children with chronic illnesses have consistently shown compromised sleep quality due to caregiving demands, emotional distress, and uncertainty (Koyuncu et al., 2022; Smith et al., 2022a). In our sample, parents also reported experiencing their own physical and cognitive symptoms, suggesting a dual burden of caregiving demands and personal health challenges that may further compromise sleep and wellbeing, especially among the mothers.

Notably, sleep problems in our sample did not significantly differ between mothers and fathers, indicating that both genders are equally susceptible to sleep disruptions in the context of caregiving for a child with long COVID. This contradicts some pre-pandemic literature where mothers typically reported higher sleep burden, such as fragmented sleep, shorter longest consecutive sleep duration, and more nocturnal awakenings (Boergers et al., 2007). One possible explanation is that the prolonged and unpredictable nature of long COVID symptoms necessitates a redistribution of caregiving responsibilities, with fathers assuming more active roles, particularly when mothers themselves are affected by post-viral symptoms. Alternatively, the expectation for both parents to maintain occupational and domestic functioning despite high caregiving demands may contribute to a more uniform sleep disruption across genders.

Children in this study reported similarly poor sleep quality (95% scoring above cut-off), which echoes previous findings among pediatric long COVID cohorts. Brackel et al. (2021) and Buonsenso et al. (2022) found that sleep disruptions in long COVID-affected children are common and often persist for months. However, the lack of statistically significant correlations between sleep measures in parent–child dyads suggests that individual experiences of sleep disruption may not be tightly coupled, despite shared household stressors.

Although sleep quality and insomnia severity appear similar at the group level, the lack of dyadic concordance indicates that sleep difficulties vary independently within families. This underscores the importance of considering individual health status, coping mechanisms, and role expectations when addressing sleep-related outcomes in long COVID-affected households.

Third, parents’ persistent symptoms were associated with worse sleep quality, more severe insomnia. While both persistent symptoms and caregiving demands likely contribute to sleep problems, our data cannot fully disentangle these effects. Interestingly, parents who reported persistent symptoms after COVID-19 had significantly higher self-esteem compared to those without persistent symptoms, which contrasts with our initial assumption that ongoing health problems would lower self-esteem. One possible explanation is that parents experiencing their own symptoms may adopt coping strategies or perceive themselves as resilient in managing dual challenges, which could enhance their sense of self-worth. Previous research suggests that problem-focused and emotion-focused coping generally supports self-esteem and well-being, whereas avoidant coping tends to have the opposite effect (Chung et al., 2023; Goodday et al., 2019). Encouraging adaptive coping and self-compassion may therefore be important components of interventions aimed at supporting parents’ mental health and self-esteem alongside physical health care.

The most striking psychological finding is the low average self-esteem among parents, which falls well below the normative threshold. This supports the theoretical link between chronic caregiving, emotional exhaustion, and diminished self-worth (Unvanli et al., 2023; Wang and Anderson, 2018). Parents reported frequent feelings of worry, particularly about their child’s schooling (69%) and future prospects (46%), highlighting the emotional labor involved.

Children reported significantly higher self-esteem than their parents, despite the shared family context, there was no consistent relationship between individual parent–child dyads. This suggests that self-esteem may be influenced by different mechanisms in parents and children, where this discrepancy may reflect developmental resilience among adolescents, or alternatively, a lack of insight into the long-term implications of their condition. However, the absence of correlations within dyads again points to divergent influences shaping self-esteem across generations. Angelhoff et al. (2021) similarly found that self-esteem in parent–child dyads may be loosely linked, especially under psychological strain, such as bereavement or chronic illness.

Taken together, these findings underscore the complexity of psychological adaptation in families affected by long term illness. While parents appear to internalize the chronic stress associated with caregiving, children may be buffered by developmental factors or social support systems. This divergence highlights the need for tailored psychosocial interventions that address both individual and relational dimensions of well-being.

### 4.1 Clinical implications

The high prevalence of insomnia and poor sleep quality among parents has clear implications for clinical practice. As others have suggested (Cerri et al., 2023; Souza et al., 2021), validated tools such as the PSQI and ISI should be routinely employed in pediatric care settings when evaluating family needs. This would enable timely identification and intervention for at-risk caregivers.

Multidisciplinary interventions are critical. Cognitive behavioral therapy for insomnia (CBT-I), sleep hygiene education, and, where appropriate, pharmacological support, should be considered. These individual interventions need to be complemented by psychological support, such as family counseling and stress management. Parent stress has been shown to directly affect child recovery and well-being (Gassman-Pines et al., 2020). Given the symptom burden among some parents, access to adult long COVID clinics should be integrated into pediatric pathways when needed. A holistic, family-centered care model, that supports both the child and symptomatic caregivers reflects real-world needs. To develop such interventions, it is crucial to understand how long COVID affects family dynamics, including psychological, emotional, and social aspects, as well as caregiving strategies and health interactions within households. Another aspect is preparedness for the next pandemic. The experience from Covid-19 shows that it is not only the initial mortality and morbidity that takes its toll on individuals, families and society, but also the long-term effects must be considered. It can be recommended to integrate this knowledge into plans to mitigate effects of coming pandemics.

### 4.2 Research implications

Future research should adopt longitudinal designs to track the evolution of parent and child well-being over time, especially as long COVID symptoms wax and wane. Larger, more diverse samples would increase the generalizability of findings and allow for subgroup analysis by socioeconomic status, ethnicity, and severity of symptoms. Importantly, qualitative research may deepen our understanding of lived experiences, particularly around coping strategies, identity changes, and health system navigation. Integrating such insights with quantitative findings could inform more responsive, empathetic healthcare frameworks. To date, there is a lack of longitudinal studies that follow children with long COVID and simultaneously include parents’ outcomes. This gap underscores the need for future research that adopts a family perspective to better understand long-term psychosocial and health impacts.

### 4.3 Limitations and future implications

This study has several limitations that should be considered when interpreting the findings. First, the use of a cross-sectional design precludes any conclusions about causality or changes over time, as associations were measured at a single point. Second, the absence of a comparison group for both parents and children (e.g., families without long COVID) limits the ability to determine whether the observed outcomes are specific to long COVID or reflect more general caregiving challenges or child health issues. This is an important consideration, as without such a control group, the results cannot be attributed solely to having a child with long COVID. In addition, including a parent comparison group was not ethically feasible because the questionnaire focused on experiences during the child’s illness and was therefore inappropriate for parents of healthy children.

Third, the sample was drawn from two regions in Sweden, which may restrict the generalizability of the results to other geographical areas or cultural contexts. Fourth, selection bias cannot be ruled out, as only children referred to specialist clinics were included, potentially representing those with more severe symptoms. Fifth, families who did not speak Swedish were excluded, which may have reduced the diversity of the sample and limited the representation of certain socio-economic or cultural groups. Finally, data were collected through self-reported questionnaires, which may be subject to recall bias and social desirability bias.

Despite these limitations, this study has important strengths. This is one of the few studies to explore the psychosocial and sleep-related impacts of long COVID on both children and their parents using validated instruments. The integration of multiple standardized measures (PSQI, ISI, RSE) alongside a study-specific questionnaire allowed for a comprehensive assessment of symptoms and well-being. Another strength is that in families where both parents participated, responses were analyzed individually. Data checks showed that parents in the same family often reported different experiences and outcomes, indicating that they answered independently. This strengthens the validity of the findings by capturing each parent’s unique perspective rather than assuming shared variance. Furthermore, the study was embedded within a larger interdisciplinary research project, ensuring a robust clinical context and systematic recruitment procedures. These strengths provide a valuable foundation for future longitudinal and interventional research in this area.

## 5 Conclusion

To conclude, this study highlights the substantial psychosocial burden experienced by parents of children with long COVID, including persistent symptoms, poor sleep, and low self-esteem. Although children also reported poor sleep and reduced self-esteem, the lack of dyadic concordance in sleep and psychological measures suggests that individual factors rather than shared family stressors play a central role in shaping outcomes. These findings underscore the need for individualized support strategies that consider both the child’s condition and the parent’s health and caregiving capacity, while also addressing psychosocial needs across generations within affected families.

## Data Availability

The raw data supporting the conclusions of this article will be made available by the authors, without undue reservation.
